# Supplementary material for: Hilab System Device in an Oncological Hospital: A New Clinical Approach for Point of Care CBC Test, Supported by the Internet of Things and Machine Learning
Source: Diagnostics (Basel). 2023 May 11;13(10):1695. doi: 10.3390/diagnostics13101695 (PMC10217552; doi:10.3390/diagnostics13101695)

Supplementary Figure S1 - Histogram plot of the frequency distributions of all CBC analytes, evaluated in the method comparison study (n = 550 patients/group). Red graphs represent the analytes related to red blood cells (RBC, HB, HT, MCV, MCH, MCHC), yellow graphs to platelets (PLT), and blue graphs to white blood cells (WBC, NEU, LIN, MON, EOS/BAS).

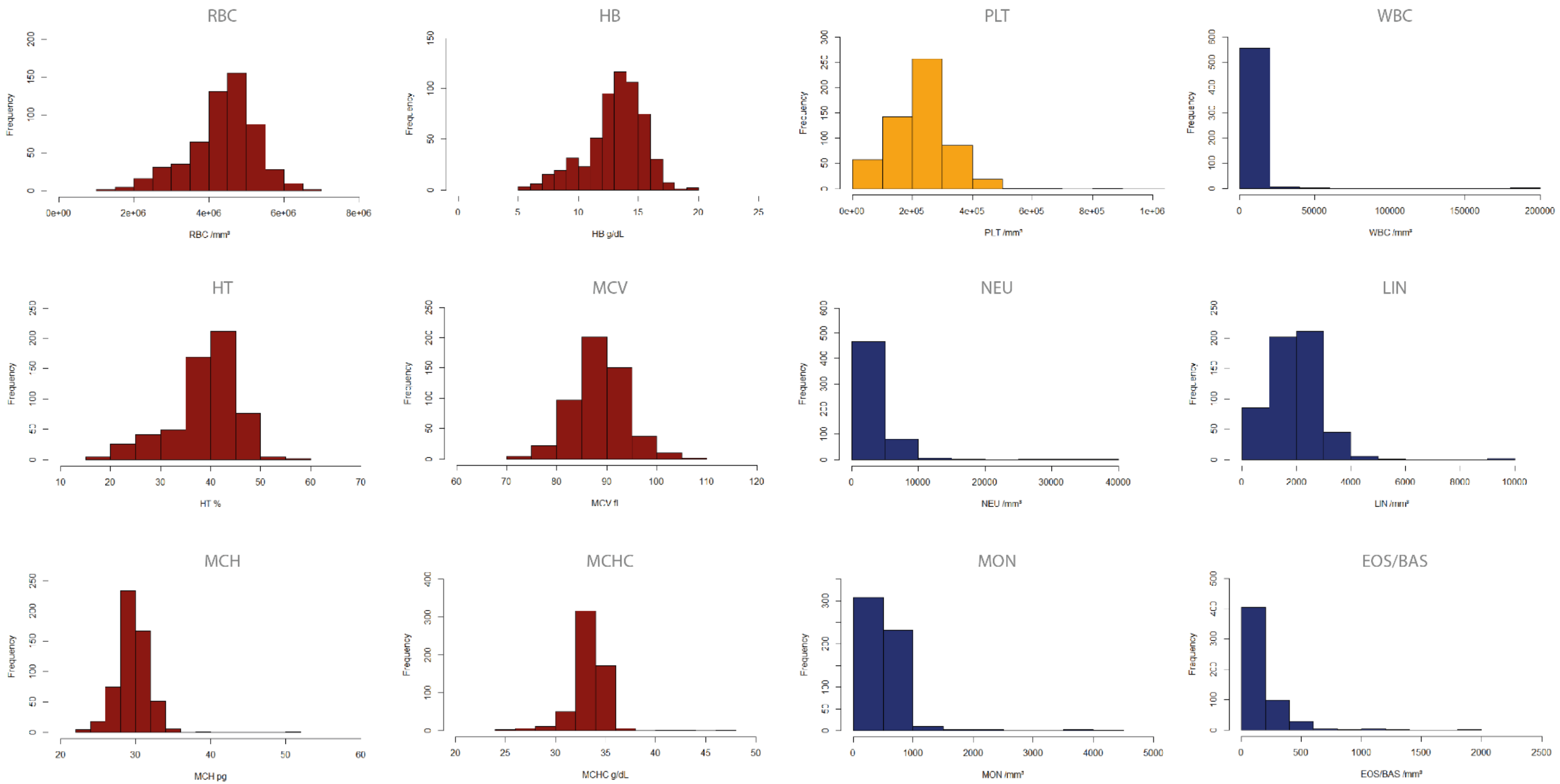

Table S1 - Descriptive statistics for method comparison study.

CBC Analytes	Min	Max	Mean	Median	Standard Deviation	Reference values[12]
RBC	1.4×10^6 /mm³	6.7×10^6 /mm³	4.4×10^6 /mm³	4.5×10^6 /mm³	827.5 $\times 10^3$ /mm³	3.8 - 5.5×10^6/mm³
HB	5.4 g/dL	19.3 g/dL	12.93 g/dL	13.21 g/dL	2.34 g/dL	12.0 - 17.0 g/dL
HT	16.8%	58.8%	38.53%	40.10%	6.56%	36.0 - 50.0%
MCV	54.5 fL	109.2 fL	88.08 fL	88.27 fL	5.95 fL	83.0 - 101.0 fL
MCH	23.6 pg	38.5 pg	29.42 pg	29.8 pg	2.15 pg	27.0 - 32.0 pg
MCHC	24.13 g/dL	46.10 g/dL	33.45 g/dL	33.68 g/dL	1.67 g/dL	31.0 - 36.0 g/dL
PLT	900 /mm³	124×10^4 /mm³	241 $\times 10^3$ /mm³	245 $\times 10^3$ /mm³	112.6 $\times 10^3$ /mm³	150 - 410×10^3 /mm³
WBC	70 /mm³	180×10^3 /mm³	6.8×10^3 /mm³	6.1×10^3 /mm³	8.8×10^3 /mm³	4 - 10×10^3 /mm³
NEU	167 /mm³	159×10^3 /mm³	3.7×10^3 /mm³	3.4×10^3 /mm³	2.9×10^3 /mm³	2 - 7×10^3 /mm³
MON	33 /mm³	5100/mm³	0.5×10^3 /mm³	0.47×10^3 /mm³	0.3×10^3 /mm³	0.2 - 1×10^3 /mm³
LIN	100 /mm³	17360/mm³	1.8×10^3 /mm³	1.8×10^3 /mm³	1.1×10^3 /mm³	1 - 3×10^3 /mm³
EOS/BAS	0/mm³	1800 /mm³	0.16×10^3 /mm³	0.14×10^3 /mm³	0.18×10^3 /mm³	0.04 - 0.6×10^3 /mm³

Supplementary Figure S2 - Bland–Altman plot of the method comparison study between the Hilab System and the Sysmex XE-2100. Bias, Student T-test p-value, upper limit of agreement (ULA), and lower limit of agreement (LLA) are demonstrated for each analyte.

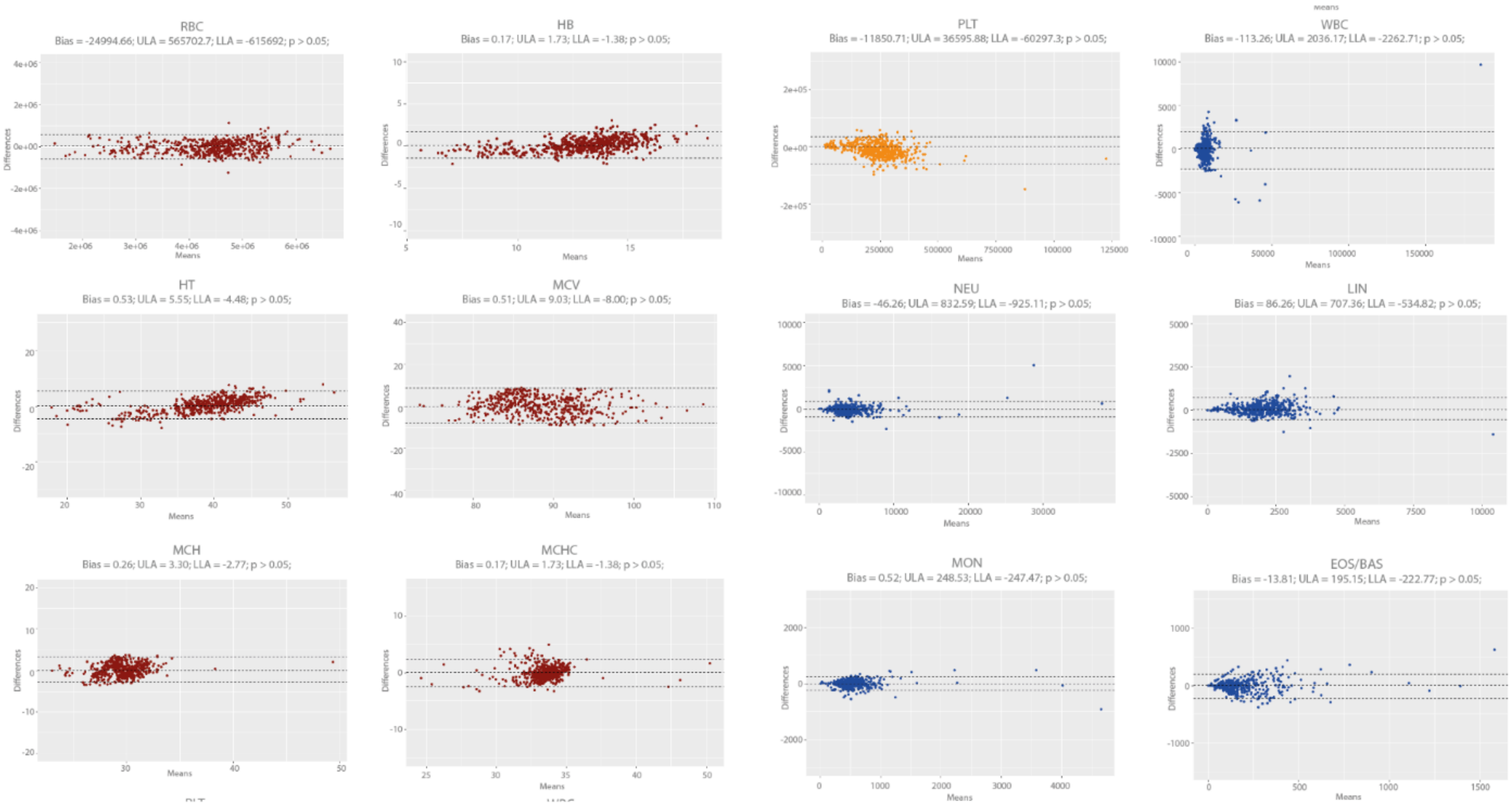

Table S2 - Descriptive statistics for the equivalence study between venous and capillary blood samples.

CBC Analytes	Min	Max	Mean	Median	Standard Deviation	Reference values
RBC	$2.6x10^6$ /mm³	$6.2x10^6$ /mm³	$4.68x10^6$ /mm³	$4.71x10^6$ /mm³	$656x10^3$ /mm³	3.8 - $5.5x10^6$/mm³
HB	7.0 g/dL	17.43 g/dL	13.6 g/dL	13.8 g/dL	1.4 g/dL	12.0 - 17.0 g/dL
HT	28.86%	50.09%	39.6%	39.78%	3.49%	36.0 - 50.0%
MCV	67.92 fL	122.96 fL	86.77 fL	85.8 fL	5.83 fL	83.0 - 101.0 fL
MCH	21.21 pg	38.77 pg	29.56 pg	30.0 pg	1.62 pg	27.0 - 32.0 pg
MCHC	34.13 g/dL	39.10 g/dL	33.74 g/dL	33.99 g/dL	1.2 g/dL	31.0 - 36.0 g/dL
PLT	$84x10^3$ /mm³	$604x10^3$ /mm³	$256x10^3$ /mm³	$248x10^3$ /mm³	$729x10^3$ /mm³	150 - $410x10^3$ /mm³
WBC	$1.7x10^3$ /mm³	$16.1x10^3$ /mm³	$6.9x10^3$ /mm³	$6.6x10^3$ /mm³	$2.1x10^3$ /mm³	4 - $10x10^3$ /mm³
NEU	$1.5x10^3$ /mm³	$10.9x10^3$ /mm³	$3.8x10^3$ /mm³	$3.5x10^3$ /mm³	$1.4x10^3$ /mm³	2 - $7x10^3$ /mm³
MON	$0.1x10^3$ /mm³	$1.9x10^3$ /mm³	$0.53x10^3$ /mm³	$0.47x10^3$ /mm³	$0.2x10^3$ /mm³	0.2 - $1x10^3$ /mm³
LIN	$0.5x10^3$ /mm³	$6.3x10^3$ /mm³	$2.2x10^3$ /mm³	$2.1x10^3$ /mm³	$0.9x10^3$ /mm³	1 - $3x10^3$ /mm³
EOS/BAS	$0.03x10^3$ /mm³	$1.1x10^3$ /mm³	$0.19x10^3$ /mm³	$0.13x10^3$ /mm³	$0.16x10^3$ /mm³	0.04 - $0.6x10^3$ /mm³

Supplementary Figure S3 - Histogram plot of the frequency distributions of all CBC analytes, evaluated in the equivalence study between venous and capillary blood samples (n = 150 patients/group). Red graphs represent the analytes related to red blood cells (RBC, HB, HT, MCV, MCH, MCHC), yellow graphs to platelets (PLT), and blue graphs to white blood cells (WBC, NEU, LIN, MON, EOS/BAS).

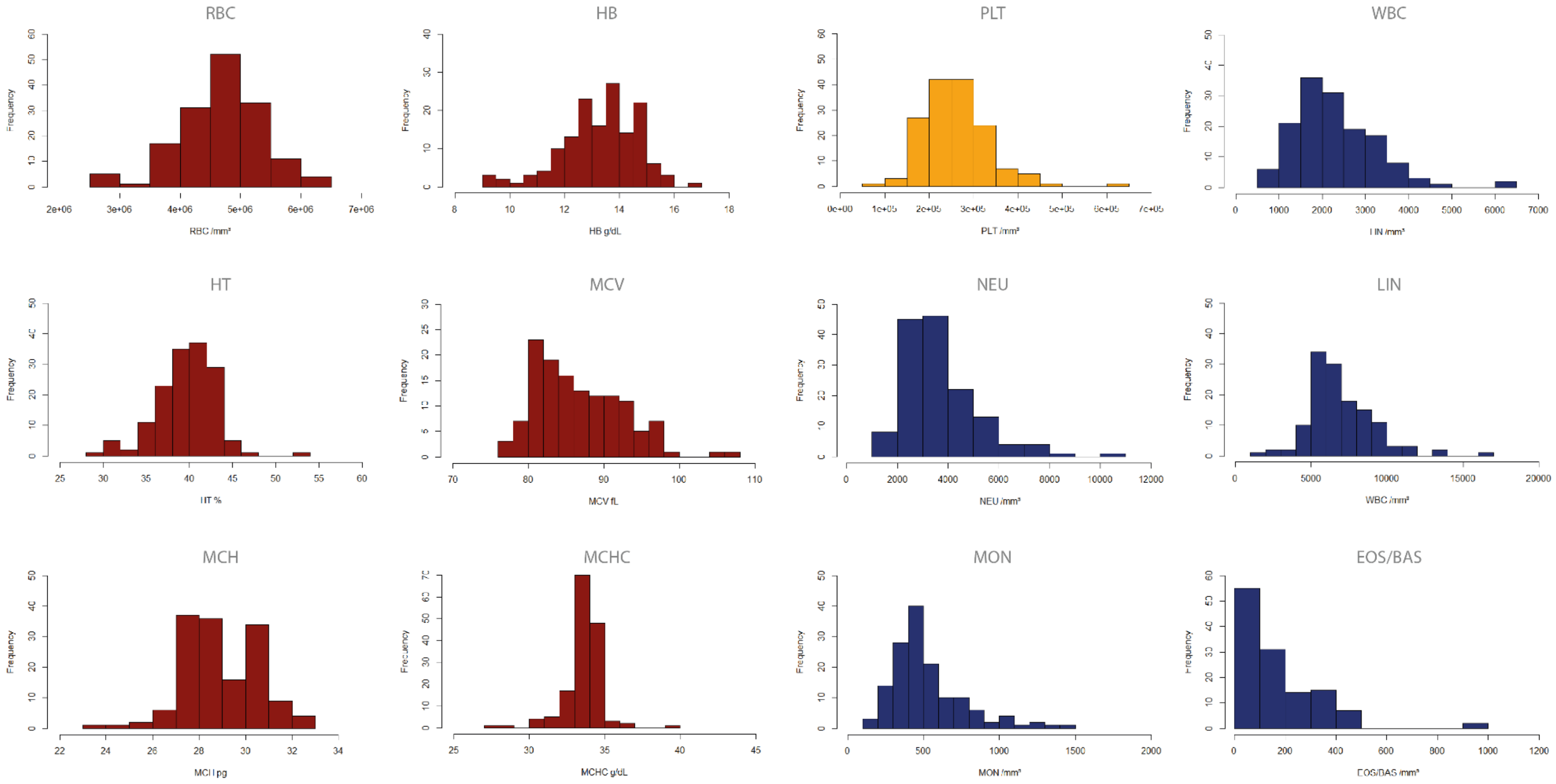

Supplementary Figure S4 - Graphical illustration of the Hilab Lens machine learning model, based on continually refining and updating processes.

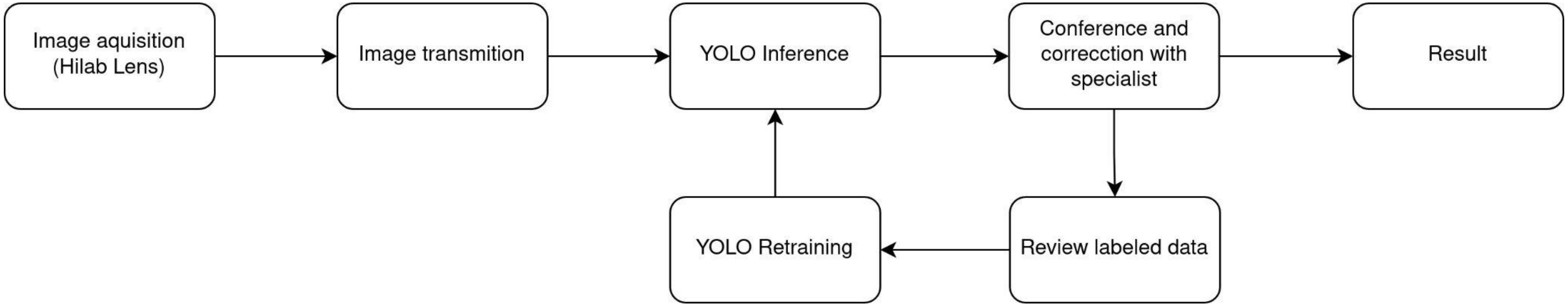

Supplement: Supplementary file 1 [file diagnostics-13-01695-s001.zip › diagnostics-2365896-supplementary.pdf]
